# Comparative genetics of invasive populations of walnut aphid, *Chromaphis juglandicola*, and its introduced parasitoid, *Trioxys pallidus*, in California

**DOI:** 10.1002/ece3.3667

**Published:** 2017-12-07

**Authors:** Jeremy C. Andersen, Nicholas J. Mills

**Affiliations:** ^1^ Department of Environmental Science Policy and Management University of California Berkeley Berkeley CA USA

**Keywords:** agroecosystems, biological control, gene flow, geographic mosaic of coevolution, inbreeding

## Abstract

Coevolution may be an important component of the sustainability of importation biological control, but how frequently introduced natural enemies coevolve with their target pests is unclear. Here we explore whether comparative population genetics of the invasive walnut aphid, *Chromaphis juglandicola,* and its introduced parasitoid, *Trioxys pallidus*, provide insights into the localized breakdown of biological control services in walnut orchards in California. We found that sampled populations of *C. juglandicola* exhibited higher estimates of genetic differentiation (*F*_ST_) than co‐occurring populations of *T. pallidus*. In contrast, estimates of both the inbreeding coefficient (*G*_IS_) and contemporary gene flow were higher for *T. pallidus* than for *C. juglandicola*. We also found evidence of reciprocal outlier loci in some locations, but none showed significant signatures of selection. *Synthesis and applications*. Understanding the importance of coevolutionary interactions for the sustainability of biological control remains an important and understudied component of biological control research. Given the observed differences in gene flow and genetic differentiation among populations of *T. pallidus* and *C. juglandicola*, we suspect that temporary local disruption of biological control services may occur more frequently than expected while remaining stable at broader regional scales. Further research that combines genomewide single nucleotide polymorphism genotyping with measurements of phenotypic traits is needed to provide more conclusive evidence of whether the occurrence of outlier loci that display significant signatures of selection can be interpreted as evidence of the presence of a geographic mosaic of coevolution in this system.

## 1 INTRODUCTION

The potential for species interactions to drive the evolution of adaptations and counter‐adaptations has been widely recognized since the seminal work of Ehrlich and Raven (1964), and has been expanded upon by the pioneering works of Janzen (1966) and Thompson (2009a). However, empirical evidence for reciprocal selection and coadaptation remains limited and best represented among species with antagonistic interactions (Carmona, Fitzpatrick, & Johnson, 2015). In this context, the conflict between insect hosts and their parasitoids is of particular interest as the interaction involves the death of one of the two participants, and thus, strong reciprocal selection might be expected to result in dynamic coevolution (Abrams, 2000; Dupas, Carton, & Poirie, 2003; Fors, Markus, Theopold, Ericson, & Hambäck, 2016; Kraaijeveld, Van Alphen, & Godfray, 1998). Both variations in host resistance to parasitism and in parasitoid counter resistance have been documented for *Drosophila melanogaster* Meigen and its parasitoids (Kraaijeveld & Godfray, 1999, 2009; Lynch, Schlenke, & de Roode, 2016), and the influence of defensive symbionts on the antagonistic coevolution of aphids and their parasitoids has been particularly well studied (e.g., Dion, Zele, Simon, & Outreman, 2011; Nyabuga, Loxdale, Heckel, & Weisser, 2012; Oliver, Russell, Moran, & Hunter, 2003; Rouchet & Vorburger, 2014; Schmid, Sieber, Zimmerman, & Vorburger, 2012; Vorburger, 2014, 2017). However, we have surprisingly little knowledge of how genetic variation influences host–parasitoid coevolution in the field (but see Henter, 1995; Henter & Via, 1995; and Fors et al., 2016).

One of the key difficulties in finding evidence for coevolution is the identification of the ecologically relevant traits or genes that are under reciprocal selection in antagonistic interactions. Recently, population genomics has been utilized to facilitate our understanding of evolutionary (Black et al. 2001, Luikart et al.^2003^, Stinchcombe & Hoekstra 2008; Deagle et al., 2011; Barker, Andonian, Swope, Luster, & Dlugosch, 2017) and coevolutionary processes (Parchman, Buerkle, Soria‐Carrasco, & Benkman, 2016; Vermeer, Dicke, & de Jong, 2011; Yoder, 2016). Through the examination of large numbers of neutral markers, population genomics can be used to separate locus‐specific effects that may be linked to genes under selection, from genomewide effects driven by genetic drift, migration, and inbreeding. This has the advantage that a population genomics approach can be applied to a wide variety of nonmodel organisms under field conditions. To investigate whether coevolutionary processes operate at different spatial or temporal scales in an antagonistic interaction, it is necessary to find evidence of the occurrence of geographic selection mosaics, trait remixing, and hot and cold spots of coevolution (Gomulkiewicz et al., 2007; Thompson, 2005). In this context, Vermeer et al. (2011) suggest that while population genomics cannot be used to test for the existence of a geographic mosaic of coevolution per se, it can be a valuable approach for the detection of unusual levels of variation or “outliers” at specific loci that are potential indicators of hot spots of reciprocal selection, and for estimation of the extent of gene flow and inbreeding that are factors contributing to trait remixing.

One field setting in which insect host and parasitoid coevolution is thought to play an important role (see Holt & Hochberg, 1997) is during the importation and establishment of non‐native parasitoids to suppress the abundance of invasive insect pests (Heimpel & Mills, 2017; Hoddle, 2004; Van Driesche et al., 2010). Biological control programs are known to be well suited for the study of evolution (Roderick, Hufbauer, & Navajas, 2012; Roderick & Navajas, 2003), but few studies exist that have used population genetic techniques to conduct comparative analyses of evolutionary change postintroduction. This may, in part, be explained by a predominant focus in biological control on pre‐introduction surveys for natural enemies without sufficient emphasis on longer‐term postintroduction monitoring (McCoy & Frank, 2010; Mills, 2000, 2017).

Our study system consists of walnut, an exotic tree crop in California; walnut aphid, *Chromaphis juglandicola* (Kaltenbach), an invasive species that is active from March until early December (Sluss, 1967); and *Trioxys pallidus* (Haliday), an introduced exotic parasitoid wasp. As is typical for nonhost alternating aphids, walnut aphids reproduce through cyclical parthenogenesis (Simon, Rispe, & Sunnucks, 2002), in which females produce multiple generations of female offspring asexually through the summer. In the fall, decline in photoperiod triggers the development of a single sexual generation of both male and female aphids, and oviparous females deposit eggs which overwinter until the following spring (Davidson, 1914). Walnut aphid appears not to have secondary defensive symbionts (Russell, Latorre, Sabater‐Muñoz, Moya, & Moran, 2003), and our own surveys of populations in California support this earlier observation (J.C. Andersen, unpublished data). Biparental hymenopteran parasitoids, such as *T. pallidus*, reproduce through haplodiploidy in which haploid males have a single maternally inherited copy of each chromosome and diploid females have both a paternal and maternal copy of each chromosome (Heimpel & de Boer, 2008). This wasp was originally introduced from southern France, which resulted in establishment in the southern and coastal regions, but failed to establish the parasitoid in the primary walnut growing region of the Central Valley (Schlinger, Hagen, & van den Bosch, 1960). Subsequently, a second introduction from Iran led to widespread establishment and reduction in walnut aphid densities throughout California (van den Bosch et al., 1979). While outbreaks of walnut aphids have occurred since the establishment of the Iranian strain of *T. pallidus*, these were associated with the use of azinphosmethyl, an insecticide used for the control of other walnut pests, and a resistant population of *T. pallidus* was reared and released (Brown, Cave, & Hoy, 1992; Hoy & Cave, 1989; Hoy et al., 1990). More recently, localized increases in the abundance of aphids have led to a resumption of in‐season insecticidal treatments in walnut orchards in California (Hougardy & Mills, 2008), and the reason for this remains unknown.

In a previous study, we examined whether hybridization among descendants of two different introduced populations of *T. pallidus* may have played a role in the observed breakdown of biological control services (Andersen & Mills, 2016). While hybridization was found to be rare in California, we did find evidence of genetic differentiation among populations of the introduced parasitoid. Given the genetic structuring of *T. pallidus* populations in walnut orchards in California, we were interested to know whether *C. juglandicola* populations displayed similar patterns of differentiation and how these patterns varied geographically within California. Therefore, the objectives of this study were (1) to compare levels of genetic differentiation among populations of *T. pallidus* and *C. juglandicola* in California, (2) to estimate rates of gene flow and inbreeding among these populations for both species, and (3) to use population genetics to detect outlier loci and the potential for reciprocal selection as preliminary evidence for the existence of a geographic mosaic of coevolution in the walnut aphid biological control program.

## 2 METHODS AND MATERIALS

### 2.1 Sampling locations

Californian walnut orchards were visited between 2010 and 2014. Orchards were selected to represent a broad range of geographic locations, but there was no prior information on the history of walnut aphid densities or levels of parasitism in each orchard. At each location, we collected individuals identified as *T. pallidus* either by aspirating adults or by collecting mummified walnut aphids and placing small cut‐out sections of leaf material with each mummy into glass vials (9.5 mm × 3 mm). These vials were closed with a foam stopper and stored at room temperature until adults emerged. Whether aspirated, or reared, adults of *T. pallidus* were then stored in 95% ethanol at −20°C for molecular analysis. Individuals of *C. juglandicola* were collected in the field and immediately placed in 95% ethanol and then stored at −20°C for molecular analysis. Effort was taken to collect only a few individuals per tree and to prioritize sampling from as many different trees as possible in each orchard to reduce the sampling of clonally related individuals (Lozier, Roderick, & Mills, 2007). Full details for each of the sampling locations are presented in Table 1.

**Table 1:** Collection locality and genetic summary information for populations of *Trioxys pallidus* and *Chromaphis juglandicola*

ID	Location	Host	Collector(s)	Date	*N* a	Num^b^	Eff_num^c^	*H*_o_ d	*H*_s_ e	*H*_t_ f	*G*_IS_ g
*T. pallidus*											
J0178	Yuba City	*P. juglandis*	J. Andersen	27xi2011	7	2.333	1.801	0.217	0.393	0.393	0.448
J0057	Arbuckle	*C. juglandicola*	J. Andersen	19vii2010	14	3.333	1.822	0.295	0.374	0.374	0.210
J0073	Upper Lake	*C. juglandicola*	J. Andersen and M. Labbé	13ix2010	13	2.933	1.710	0.264	0.334	0.334	0.211
J0179	Escalon	*C. juglandicola*	J. Andersen	5vi2012	12	3.067	1.833	0.265	0.352	0.352	0.245
J0188	Newark	*C. juglandicola*	J. Andersen and M. Labbé	30viii2012	10	2.867	1.869	0.347	0.389	0.389	0.107
*C. juglandicola*											
A0052	Linden	*J. regia*	J. Andersen	10vii2010	7	1.500	1.208	0.119	0.139	0.139	0.143
A0057	Arbuckle	*J. regia*	J. Andersen	19vii2010	12	2.333	1.615	0.201	0.362	0.362	0.444
A0070	Upper Lake	*J. regia*	J. Andersen and M. Labbé	13ix2010	9	1.667	1.288	0.179	0.197	0.197	0.089
A0046	Escalon	*J. regia*	J. Andersen and K. Anderson	7vii2010	9	1.500	1.152	0.102	0.097	0.097	−0.048
A0189	Newark	*J. regia*	J. Andersen and M. Labbé	18ix2014	12	1.833	1.259	0.160	0.167	0.167	0.043

a Number of samples.

b Average number of alleles across microsatellite markers.

c Average effective number of alleles across microsatellite markers.

d Observed heterozygosity.

e Heterozygosity within populations.

f Total heterozygosity.

g Inbreeding coefficient.

### 2.2 DNA extraction and microsatellite genotyping

DNA was extracted from adult females of *T. pallidus* and *C. juglandicola* by grinding individuals with a mortar and pestle and then using the modified DNA extraction protocols presented in Andersen and Mills (2014). Standard PCR protocols were then used to amplify 15 polymorphic microsatellite markers for *T. pallidus* and 12 polymorphic microsatellite markers for *C. juglandicola* following protocols presented in Andersen and Mills (2014). Briefly, microsatellite loci were amplified from 7 to 20 aphids and/or parasitoid females from each location using fluorescently labeled primers, and PCR products for up to four loci were pooled before genotyping so that no two loci with the same fluorescent label were combined. Products were then genotyped on an Applied Biosystems 3730XL DNA Analyzer at the University of California Berkeley DNA Sequencing Facility using the LIZ 600 size standard, and fragment lengths were then scored using the Microsatellite Plug‐in for Geneious Pro v. 5.6.2 (Drummond et al., 2012).

### 2.3 Population genetic analyses

For each population, standard population genetic statistics including the average number of alleles per locus (Num), the average effective number of alleles per locus (Eff_num), the observed heterozygosity (*H*_o_), within‐population heterozygosity (*H*_s_), total heterozygosity (*H*_t_), and the inbreeding coefficient (*G*_IS_) were all estimated using the software program GenoDive v.2.0b27 (Meirmans & Van Tienderen, 2004). Departures from Hardy–Weinberg equilibrium (HWE) and the presence of locus‐by‐locus linkage disequilibrium (LD) were then estimated with the software package GenePop (Raymond & Rousset, 1995; Rousset, 2008). Estimates of population differentiation based on *F*_ST_ were generated using FreeNA (Chapuis & Estoup, 2007) to account for the potential presence of null‐alleles, and whether populations were significantly differentiated between each population pair was determined using the exact G test implemented in GenePop.

Recent migration rates (i.e., the proportion of individuals in a population that were estimated to be derived from a second population) were then estimated between each population using the BayesAss Edition v. 3.0 (BA3) software package (Wilson & Rannala, 2003). Four independent analyses for each species were conducted, each using a mixing parameter of 0.8 for allele frequencies, migration rates, and inbreeding coefficients and a runtime of 10 million generations with a burn‐in period of 1 million generations. Results were then visualized and summarized across runs for each species using the program Tracer v. 1.6.0 (Rambaut & Drummond, 2007).

### 2.4 Geographic mosaic of coevolution

Recently, Vermeer et al. (2011) presented a population genomics approach to investigate the potential for geographic mosaics of coevolution. Based on this approach, neutral genetic loci for two (or more) interacting species are used to survey individuals at a number of geographic locations where both species co‐occur as well as a single location for each species where its counterpart is absent. Using the latter as known coevolutionary cold spots, pairwise comparisons of genetic diversity (e.g., *F*_ST_ *, G*_ST_ *,* Rho_ST_, etc.) between these and other locations can be estimated for each locus and used to look for outlier loci. If outliers are detected at a particular location for both interacting species, the location may be a coevolutionary hot spot, whereas if outliers are present for only one or neither of the interacting species, the location may be a coevolutionary cold spot. These same neutral loci can then be used to estimate levels of gene flow between locations. Finally, evidence for population structure (genetic diversity) and outlier loci can be used to identify potential hot and cold spot locations, which in conjunction with measurements of phenotypic or behavioral traits, and can be used to confirm whether the interacting species are under reciprocal selection. By combining measures of gene flow and inbreeding with outlier detection using both known cold spot and undetermined hot and cold spot locations, this approach addresses the three underlying processes that form the basis for testing the geographic mosaic theory of coevolution; (1) coevolutionary hot and cold spots, (2) selection mosaics, and (3) trait remixing (Gomulkiewicz et al., 2007).

Following this approach, we utilized two of our sampled orchards from which only one of the two interacting species was present to act as our reference coevolutionary cold spot locations. The reference cold spot for *T. pallidus* was located near Yuba City, CA where we found *T. pallidus* parasitizing an alternative host, the dusky‐veined aphid, *Panaphis juglandis* (Goeze) (Hemiptera: Aphididae). The reference cold spot for our *C. juglandicola* analyses was located near Linden, CA, where we found walnut aphids, but were unable to locate any adult *T. pallidus* or aphid mummies. To identify outliers, we then estimated locus‐specific measures of genetic differentiation for both *C. juglandicola* and *T. pallidus* using our above null‐allele estimates of *F*_ST_ and Rho_ST_ in GenePop based on pairwise comparisons between the reference cold spots and each candidate location. Using the “boxplot” function in the statistical software package R v. 3.1.3 (R Core Team 2015), we then visually examined whether the distribution of the locus‐specific estimates for each location and outlier loci was detected by falling outside of the whiskers representing 1.5 times the interquartile range. To test whether any of these outliers were statistically significant, we utilized the program BayeScan v. 2.1 (Foll & Gaggiotti, 2008) using the default settings to conduct reversible‐jump MCMC simulations. While BayeScan has been shown to have a higher false discovery rate than other tests under complex demographic scenarios (Hoban et al., 2016; de Villemereuil, Frichot, Bazin, François, & Gaggiotti, 2014), particularly when admixed individuals are present in the sample (Luu, Bazin, & Blum, 2017), it has been widely used for the analysis of microsatellite datasets.

## 3 RESULTS

### 3.1 Population genetic analyses

Genotyping results for the individuals analyzed are available in Appendix S1. Observed heterozygosity (mean *H*_o_ of 0.278 for *T. pallidus* and 0.152 for *C. juglandicola*) was notably low (Table 1). In addition, the inbreeding coefficient (*G*_IS_) for *T. pallidus* populations (mean ± *SD*; 0.244 ± 0.125) was considerably higher than that for *C. juglandicola* populations (mean ± *SD*; 0.134 ± 0.187), although these differences were not significant based on post hoc *t* test analyses as implemented in R (*t *=* *−1.09, *df* = 6.99, *p *=* *.31). Of the four populations from which both *T. pallidus* and *C. juglandicola* were both collected, three of these (Escalon, Newark, and Upper Lake) had higher estimates of *G*_IS_ for *T. pallidus* than for *C. juglandicola*. For *T. pallidus*, all but one population (Newark) displayed significant deviations from HWE (*p *<* *.017), whereas for *C. juglandicola* only a single population (Arbuckle) displayed significant deviations from HWE (*Χ* ^2^ = ∞, *df* = 20, *p *<* *.001).

Three of the 105 pairwise LD comparisons for the microsatellite loci amplified from *T. pallidus* (*Tp*_MSAT 4 and *Tp*_MSAT 17 [*Χ* ^2^ = 19.38, *df* = 10, *p *=* *.036], *Tp_*MSAT8 and *Tp_*MSAT19 [*Χ* ^2^ = 17.13, *df* = 8, *p *=* *.029]; *Tp_*MSAT13 and *Tp_*MSAT17 [*Χ* ^2^ = 20.52, *df* = 10, *p *=* *.025]), and five of the 66 pairwise LD comparisons for the microsatellite loci amplified from *C. juglandicola* (*Cj*_MSAT5 and *Cj*_MSAT8 [*Χ* ^2^ = 12.02, *df* = 2, *p *=* *.003], *Cj*_MSAT5 and *Cj*_MSAT9 [*Χ* ^2^ = 11.27, *df* = 4, *p *=* *.024]; *Cj_*MSAT5 and *Cj_*MSAT19 [*Χ* ^2^ = 12.02, *df* = 4, *p *=* *.017]; *Cj*_MSAT8 and *Cj_*MSAT19 [*Χ* ^2^ = 15.05, *df* = 2, *p *<* *.001]; and *Cj*_MSAT14 and *Cj*_MSAT18 [*Χ* ^2^ = 13.03, *df* = 4, *p *=* *.011]) showed a significant presence of LD. However, only the pairwise comparison between *Cj_*MSAT8 and *Cj*_MSAT19 displayed significant LD after Bonferroni's correction for multiple comparisons (corrected α for *T. pallidus* = 0.0005; corrected α for *C. juglandicola* = 0.0008).

**Figure 1:**
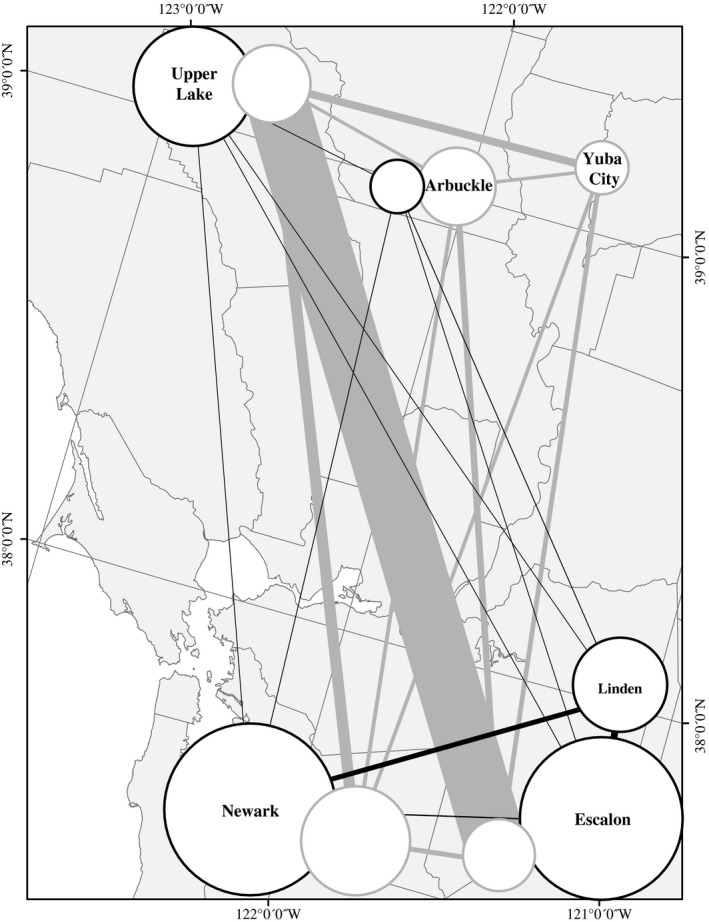
Sampling locations for California populations of *Trioxys pallidus and Chromaphis juglandicola*. The area of circles for each population (gray = *T. pallidus* and black = *C. juglandicola*) is inversely proportional to estimates of *G*_IS_ for that species at each location (values for *G*_IS_ are presented in Table 1). Circles for both species are drawn side‐by‐side at each locality with the larger circle approximately centered on the sample location. Lines connecting populations have widths inversely proportional to global estimates of *F*_ST_ (corrected for the presence of null‐alleles) for each species colored as above (values for *F*_ST_ are presented in Table 2). Geographic representations were generated in arcMap v.10.3.1 (ESRI, Redlands, CA) and visualized using the North American Albers Equal Area Conic projection

Populations of both *T. pallidus* and *C. juglandicola* showed evidence of significant population differentiation (Table 2). For *T. pallidus,* the Yuba City population was significantly differentiated from the Arbuckle and Escalon populations (although neither of these differences were significant after applying Bonferroni's correction for multiple comparisons, adjusted α = 0.005). In contrast, for *C. juglandicola,* seven of the ten pairwise comparisons showed evidence for significant differentiation of populations (all seven were significant after applying Bonferroni's correction for multiple comparisons, adjusted α = 0.005). For *T. pallidus*, there were no clear patterns of geographic structuring, as one population from the northern end of the sampled region (Upper Lake) was more similar to a population from the southern end of the sampled region (Escalon) than it was to more geographically proximal locations. However, for *C. juglandicola,* populations in the southern end of the sampled region (Escalon, Linden, and Newark) were more similar to each other (Table 2, Figure 1) than they were to those from the northern end of the sampled region (Arbuckle, Upper Lake).

**Table 2:** Measures of population differentiation among locations for *Chromaphis juglandicola* and *Trioxys pallidus* based on *F*_ST_ (lower diagonal) and *p*‐values for pairwise exact *G* tests (upper diagonal). Values in bold represent statistically significant differences (P < 0.05)

	Yuba City	Arbuckle	Upper Lake	Escalon	Newark
*T. pallidus*					
Yuba City		**0.040**	0.409	**0.040**	0.180
Arbuckle	0.036		0.084	0.074	0.075
Upper Lake	0.016	0.038		0.929	0.590
Escalon	0.025	0.019	0.002		0.101
Newark	0.033	0.031	0.011	0.024	

	Linden	Arbuckle	Upper Lake	Escalon	Newark
*C. juglandicola*					
Linden		**0.001**	**<0.001**	0.861	0.880
Arbuckle	0.137		**<0.001**	**<0.001**	**<0.001**
Upper Lake	0.295	0.209		**<0.001**	**<0.001**
Escalon	0.018	0.190	0.329		0.579
Newark	0.025	0.152	0.316	0.093	

Estimates of contemporary gene flow (i.e., migration rates) varied between population pairs for both of the interacting species; however, gene flow was only significant (95% CI's not including 0) for populations of *T. pallidus* (Table 3). There were no examples of reciprocal gene flow among population pairs, with Upper Lake having the largest number of connections (gene flow to Arbuckle, Modesto, and Yuba City, and gene flow from Newark), and Arbuckle having no significant connections.

**Table 3:** Mean measures of recent migration rates for *Trioxys pallidus* and *Chromaphis juglandicola* using BA3^a^

	Yuba city	Arbuckle	Upper Lake	Escalon	Newark
*T. pallidus*					
Yuba city		0.018	0.015	0.014	0.022
Arbuckle	0.023		0.015	0.015	0.023
Upper Lake	**0.135**	**0.191**		**0.211**	**0.179**
Escalon	**0.130**	0.089	**0.128**		0.087
Newark	0.023	0.018	0.015	0.015	

	Linden	Arbuckle	Upper Lake	Escalon	Newark
*C. juglandicola*					
Linden		0.054	0.029	0.077	0.076
Arbuckle	0.028		0.024	0.024	0.030
Upper Lake	0.029	0.024		0.025	0.022
Escalon	0.173	0.083	0.038		0.184
Newark	0.023	0.018	0.015	0.015	

a Average migration rates between each population pair should be read as Row Name→Column Name. Significant migration rates (i.e., mean migration rate ± 1.96 * standard deviation not including zero) are highlighted in bold.

### 3.2 Detection of outlier loci

**Figure 2:**
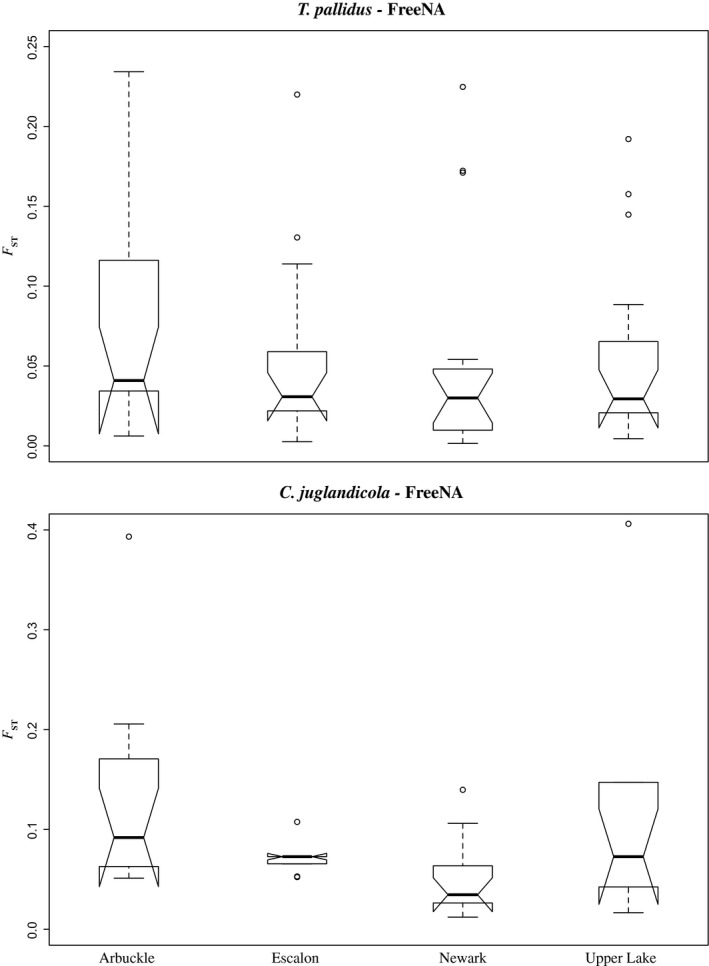
Locus‐specific measures of *F*_ST_ corrected for the presence of null‐alleles for different California populations of *Trioxys pallidus* and *Chromaphis juglandicola*. Within box plots, the dark line represents the median measure for each population (compared to the species‐specific reference population), the open box is the interquartile range (IQR), whiskers extend to 1.5 * IQR, diagonal “notches” represent 95% confidence intervals (mean ± 1.96 * standard deviation), and outlier loci are represented as open circles

Analyses based on identifying outliers from values of null‐allele corrected *F*_ST_ for each population pair using the “boxplot” function in R identified outliers in three of the pairwise comparisons for *T. pallidus* (Escalon, Newark, and Upper Lake), and in all four comparisons for *C. juglandicola* (Arbuckle, Escalon, Newark, and Upper Lake) (Figure 2). For *T. pallidus*, three loci were identified as potential outliers in two of the four pairwise comparisons (*Tp*_MSAT5; Newark and Upper Lake: *Tp_*MSAT14; Escalon and Upper Lake: *Tp*_MSAT17; Newark and Upper Lake) and two loci were outliers in one of the four pairwise comparisons (*Tp_*MSAT12; Newark: *Tp_*MSAT13; Escalon). For *C. juglandicola,* two loci were identified as potential outliers in two of the four pairwise population comparisons (*Cj*_MSAT8; Escalon and Newark: *Cj*_MSAT16; Arbuckle and Upper Lake) and three loci were identified as potential outliers in one of the four pairwise comparisons (*Cj*_MSAT1; Upper Lake: *Cj*_MSAT3; Escalon: *Cj*_MSAT4; Escalon). Using the approach of Vermeer et al. (2011), results suggested that Escalon, Newark, and Upper Lake potentially represent coevolutionary hot spots, while Arbuckle, Linden, and Yuba City potentially represent coevolutionary cold spots. However, Bayesian simulations using BayeScan indicated that none of the potential outliers for either species showed significant (*p *<* *.05) signatures of selection.

## 4 DISCUSSION

The study of coevolutionary interactions has not only helped to explain patterns of species diversity (Ehrlich & Raven, 1964; Janzen 1966; Bernays & Graham, 1988; Hembry, Yoder, & Goodman, 2014; Thompson, 2014), but has also contributed to our understanding of best management practices for natural resources (Carroll, 2011; Kinkel, Bakker, & Schlatter, 2011; Rammel, Stagl, & Wilfing, 2007). As such, coevolution has long been thought to play an important role in the sustainability of biological control services (Holt & Hochberg, 1997; Jones, Vanhanen, Pettola, & Drummond, 2014; Kraaijeveld & Godfray, 1999, 2009). For the biological control of *C. juglandicola* by *T. pallidus*, our results indicate that these two species differ in regard to their levels of genetic differentiation, with the former showing greater geographic structure than the latter. In addition, while we did find reciprocal outlier loci, indicative of the potential for coevolutionary hot and cold spots, none provided a significant signature of selection. Consequently, this study provides us with a curious and apparently contradictory pattern—namely that *T. pallidus* showed lower levels of genetic differentiation among populations while displaying elevated levels of inbreeding within populations compared to *C. juglandicola*.

This finding might have important implications for the coevolutionary stability of the walnut aphid biological control program. It is possible that the observed difference in levels of population differentiation may be a result of the different amounts of time each species has been present in western North America (>100 years for *C. juglandicola* versus ~50 years for *T. pallidus* [Davidson, 1914; van den Bosch et al., 1979]). Alternatively, it could also be due to differences in the rates of evolution as a result of selection and/or to differences in reproductive strategies (i.e., sexual and haplodiploid for *T. pallidus* versus cyclical parthenogenesis and diploid for *C. juglandicola*). While aphid species have shown evidence of rapid evolution in response to changes in their environment (e.g., Harmon, Moran, & Ives, 2009), a recent study of aphid–parasitoid coevolution found evidence for genetic tracking of both species (Nyabuga et al., 2012). In this latter study, the authors also found greater levels of differentiation among aphid populations compared to their parasitoids. However, in contrast to our results, they found that the aphid populations had greater inbreeding coefficients than the parasitoid populations and suspected that this arose from different metapopulation dynamics. The authors also considered that the rate of evolution of the parasitoid relative to that of its aphid host was constrained by the lag time in colonization of new patches (Nyabuga et al., 2012). Lag time may disrupt reciprocal selection (Lapchin & Guillemaud, 2005), and a difference in evolutionary rates of interacting organisms can have negative impacts on the stability of their relationships. For example, predator–prey dynamics can be negatively affected by the rapid evolution of the prey species (Yoshida, Jones, Ellner, Fussmann, & Hairston, 2003), while conversely, herbivore–plant dynamics can be negatively affected by the rapid evolution of the herbivore (Smith, de Lillo, & Amrine, 2010). However, why *T. pallidus* with its greater levels of gene flow among populations would display higher levels of *G*_IS_ is unclear as mathematical models predict that as migration rates increase among populations, local adaptation within those populations will decrease (Blanquart, Gandon, & Nuismer, 2012).

In a pioneering paper, Holt and Hochberg (1997) outlined a range of factors that could account for the evolutionary stability of natural enemy–victim interactions in biological control including metapopulations dynamics, temporal variability in selective pressures, and coevolutionary interactions. However, host resistance has been documented or is suspected to have occurred, in at least a couple of biological control programs (e.g., Goldson et al., 2014; Ives & Muldrew, 1984; Tomasetto, Tylianakis, Reale, Wratten, & Goldson, 2017). In one of these programs, the control of the larch sawfly in Canada, resistance to parasitism may have arisen due to the accidental importation and spread of pre‐adapted resistant host strains (Ives & Muldrew, 1984). For another, the control of the Argentine stem weevil in New Zealand, parasitism rates declined by nearly 50% over a 5‐year period (Goldson et al., 2014) starting exactly 7 years after parasitoid release irrespective of the actual year of introduction (Tomasetto et al., 2017). Similar to the walnut aphid biological control program, the Argentine stem weevil and its introduced parasitoid differ in their reproductive strategies (sexual for the Argentine stem weevil versus asexual for the parasitoid). Therefore, it is possible that coevolutionary interactions in biological control systems that rely on natural enemies with a reproductive strategy that differs from that of their target host may become decoupled due to different rates of evolution among the interacting species.

### 4.1 Geographic mosaic of coevolution

If as expected, coevolutionary interactions are both spatially and temporally dynamic (Torres, 2009) and are important for biological control services (Holt & Hochberg, 1997; Jones et al., 2014; Kraaijeveld & Godfray, 1999, 2009), then localized breakdowns in biological control services might be a common and transitory occurrence as predicted by the geographic mosaic theory of coevolution (Thompson, 1994, 2005). While geographic mosaics have been observed for interactions between herbivores and plants (Muola et al., 2010; Siepielski & Benkman, 2005; Vermeer, Verbaarschot, & de Jong, 2012), predators and prey (Brodie & Ridenhour, 2002), and hosts and parasites (Dixon, Craig, & Itami, 2009; Lorenzi & Thompson, 2011; Thompson, 2009b; Vergara, Lively, King, & Jokela, 2013), as of yet there are no known documented examples from the biological control literature. It has been proposed, however, that a geographic mosaic of coevolution may have played an important role in the establishment of invasive knapweeds (*Centaurea maculosa* Lamarck and *C. diffusa* Lamarck) in North America (Callaway, Hierro, & Thorpe, 2005).

Therefore, our finding from this study of the reciprocal presence of outlier loci among populations of *C. juglandicola* and *T. pallidus*, and trait remixing based on gene flow among populations, provides some of the first support for the potential of a geographic mosaic of coevolution in a classical biological control program. However, as Vermeer et al. (2011) outline, for this approach to provide more than preliminary evidence for the presence of a geographic mosaic of coevolution, genotype data from a large number of independent neutral loci (preferably single nucleotide polymorphisms [SNPs]) need be compared to phenotypic measurements from traits of interest, as has been done in other systems (e.g., Dupas, Dubuffet, Carton, & Poire, 2009; Jancek et al., 2013; Parchman, Benkman, Jenkins, & Buerkle, 2011; Parchman et al., 2016). Given that our current study includes genotype data only, and from a relatively small number of microsatellite loci, we are not yet able to determine whether the presence of outlier loci in both species is the result of reciprocal selection, or of random chance.

Consequently, understanding the importance of coevolutionary interactions for the sustainability of biological control remains an important and understudied component of biological control research. This is in part due to the fact that there have been very few long‐term, postrelease studies of biological control agents and their targets (McCoy & Frank, 2010; Mills, 2000, 2017), and that it can be difficult to identify a priori which adaptive traits to measure when studying coevolutionary interactions. In this context, comparative population genomics may provide a useful approach to obtain preliminary evidence for the presence and/or potential importance of coevolution in biological control systems. Based on our findings, we suspect that coevolution is important for the sustainability of biological control programs and that long‐term studies would likely reveal a continuum from sustained effective control when coevolutionary interactions are strong, to failures when they are weak. Under this scenario, biological control programs may experience temporary failures in effective control at a localized scale, and yet experience sustainable control at a regional or landscape scale due to connectivity and movement between local populations as predicted by the geographic mosaic theory of coevolution.

## CONFLICT OF INTEREST

None declared.

## AUTHORS’ CONTRIBUTIONS

JCA conduct the field sampling, generated the molecular data, and performed analyses. NJM oversaw the collection of data and provided laboratory support. Both authors contributed equally to the study design and the drafting of the manuscript.
